# Vibration Analysis and Vehicle Detection by MEMS Acceleration Sensors Embedded in PCC Pavement

**DOI:** 10.3390/s25092898

**Published:** 2025-05-04

**Authors:** Congyi Chang, Linghui Kong, Libin Han, Junmin Li, Shuo Pan, Ya Wei

**Affiliations:** 1 Key Laboratory of Civil Engineering Safety and Durability of China Education Ministry, Department of Civil Engineering, Tsinghua University, Beijing 100084, China; ccy23@mails.tsinghua.edu.cn (C.C.); lkongal@connect.ust.hk (L.K.); 2 Southwest United Graduate School, Kunming 650092, China; 3 Kunming International Aviation Hub Engineering Construction Headquarters, Yunnan Airport Group Co., Ltd., Kunming 650200, China; cafuc_ljmemail@163.com (L.H.); 18210657398m2@sina.cn (J.L.); 4 Beijing Key Laboratory of Traffic Engineering, Beijing University of Technology, Beijing 100124, China; panshuo@emails.bjut.edu.cn

**Keywords:** multiple pavement slabs, thresholding method, sensor placement locations, detection precision and recall, maximum vibration response

## Abstract

**Highlights:**

**What are the main findings?**

- The vibration response characteristics of multiple PCC pavement boards at different positions under the dynamic load of vehicles are obtained
- A thresholding method for vehicle detection is proposed, achieving high precision and recall in a concise manner.
- Sensor placement locations are optimized, enabling low-cost and highly effective vehicle detection with >85% precision and recall.

**What is the implication of the main finding?**

- The proposed thresholding method offers a cost-effective, real-time vehicle detection solution, simplifying data processing for road maintenance and traffic management.
- Optimal sensor placement strategies reduce deployment costs while maintaining high detection accuracy, enabling scalable infrastructure monitoring.
- This approach enhances pavement performance monitoring by eliminating the need for complex signal denoising, supporting proactive maintenance and improved traffic safety.

**Abstract:**

Monitoring the vibration response of Portland cement concrete (PCC) pavement under dynamic vehicle loading is critical for road maintenance and traffic analysis. This study embedded micro-electro-mechanical systems (MEMS) accelerometer sensors in PCC pavement to capture vibration signals induced by vehicles. A thresholding method is proposed to automate vehicle detection by analyzing acceleration time-domain data, achieving precision and recall rates exceeding 85%. The study also explored various sensor placement locations and different threshold values for acceleration time-domain signals. Sensor placement optimization revealed that positioning sensors at the front or rear ends of pavement slabs maximizes vibration response, enabling low-cost and efficient detection. Experimental results demonstrated that the proposed method balances simplicity and accuracy, eliminating the need for complex denoising processes. This approach provides a cost-effective solution for real-time vehicle detection and enhances pavement performance monitoring, supporting improved maintenance and traffic management strategies.

## 1. Introduction

Portland cement concrete (PCC) pavement is a crucial component of transportation infrastructure, renowned for its impressive structural stability and enduring durability. Composed of Portland cement, water, and aggregates like sand and gravel, PCC undergoes a chemical reaction known as hydration when the cement and water mix, leading to a hardening process that results in a strong and durable material [1]. However, variations in the strength and rigidity of cement concrete over time [2], coupled with a propensity for cracking [3,4], highlight its vulnerability. As service time extends, the performance of PCC pavement often deteriorates, leading to potential damage [5]. The dynamic loading from vehicles exacerbates this issue, inducing fatigue damage that further weakens the pavement structure [6]. Therefore, consistent monitoring of PCC pavement is vital for proactive maintenance and timely repairs [7]. Additionally, capturing vehicle information during pavement monitoring supports traffic flow analysis [8].

Sensor deployment for pavement monitoring is a widely accepted practice [9]. For instance, Dong et al. developed cement-based sensors infused with carbon nanofibers, demonstrating exceptional piezoresistive properties for detecting pedestrian and vehicle movements [10]. Braunfelds et al. utilized Fiber Bragg grating optical sensors atop cement-treated recycled asphalt pavement layers, effectively measuring road strain under dynamic vehicle loads [11]. Zhu et al. explored the efficacy of piezoelectric composite sensors in axle load detection, uncovering the significant influence of sensor position on performance, whereas burial depth showed minimal impact [12]. Additionally, sensors are applied in more traffic infrastructure monitoring fields. Liu et al. reviewed the deployment of pressure sensors in monitoring pantograph systems on high-speed railways [13]. Rizzo et al. reviewed the application of wireless sensors in the field of bridge monitoring [14].

Recently, the adoption of micro-electro-mechanical systems (MEMS) accelerometer sensors has grown due to their compact size, high efficiency, and low energy consumption, marking their utility in pavement monitoring [15,16,17]. Zhang et al. employed these sensors to gather comprehensive acceleration, rotation, and stress data. They applied algorithms like normalized cross-correlation, smoothed coherence transform, and phase transformation for accurate load speed estimation in pavement tests and real-time traffic speed monitoring, with the smoothed coherence transform algorithm showing optimal results [18]. Shi et al. embedded MEMS sensors in semi-rigid pavement, using accelerated testing to evaluate the influence of loads on vehicle speed estimation [19]. This study leveraged the benefits of MEMS accelerometer sensors for efficient data collection.

However, the signals collected by acceleration sensors often contain significant noise, which in turn affects the detection efficacy of the acceleration sensors. To reduce the noise, various denoising techniques are employed, among which the SWT (stationary wavelet transform) denoising method is increasingly applied in the denoising of non-stationary signals due to its robust performance. Qin et al. applied the SWT denoising method to the signal processing of distributed sensors and demonstrated that the SWT method can effectively reduce the noise in the signals of distributed sensors [20]. Zeng et al. embedded distributed optical vibration sensing (DOVS) sensors into the road surface to detect vehicle signals and applied the SWT method for signal denoising. They proved that the performance of the SWT method was better than both median filtering and band-pass filtering in the denoising of vehicle signals [21].

In the realm of pavement monitoring, a variety of methods have been implemented to analyze structural vibrations due to dynamic vehicle loading. Stocker et al. employed vibration sensors and machine learning for vehicle detection and classification, achieving detection accuracy of between 94% and 100%, and classification accuracy ranging from 43% to 86% [22]. Bajwa et al. designed a wireless sensor network (WSN) with acceleration sensors for reporting pavement vibrations and vehicle-detection sensors for logging vehicle arrival and departure times, alongside an access point for data synchronization and recording [23]. Addressing energy self-sustainability and data transmission cost issues in WSN-based pavement vibration monitoring, Klis et al. proposed integrating existing spectral–temporal information with novel compressive sensing techniques, fostering robust signal reconstruction and reducing transmission expenses [24]. Zhao et al. used distributed optical vibration sensors to record pavement vibrations from dynamic loads, applying empirical mode decomposition for signal reconstruction and feature extraction, aiming to discern axle configurations and vehicle speeds [25]. Ye et al. developed a printed circuit board with three MEMS accelerometer chips (VS1002, MS9001, and ADXL355). By testing the sensing performance of the three MEMS accelerometer chips, in terms of sensitivity, linearity, noise, resolution, frequency response, and temperature drift, it was found that the VS1002 had the highest sensitivity and resolution [26]. To effectively use data on temporal, spatial, and spectral vibration distributions of PCC pavement, Zeng et al. introduced a method for reconstructing and analyzing vibration fields from one-dimensional measured data, effectively reflecting traffic behaviors like braking and lane-changing [27].

Considering the structure of PCC pavement, which comprises multiple slabs connected by force-transmitting or tension rods, dynamic vehicle loading results in varied vibration responses across different slabs. Even within a single slab, the response varies across locations. These discrepancies significantly influence the performance assessment of PCC pavement and the efficiency of vehicle detection, areas that existing research has not thoroughly explored. To fill these gaps, this study strategically placed multiple MEMS accelerometer sensors on various slabs and positions of the PCC pavement. The aim was to understand the relationships between the responses of different slabs and positions under vehicle load, enhancing vehicle-detection accuracy and identifying optimal sensor placements.

Pavement’s vibration response to vehicle loading is captured as one-dimensional acceleration time-series data. Several methods are used to analyze these vibrations, each with its own benefits and limitations. Liang et al. assessed the impact of noise on road and railway vibration signals using deep-learning techniques [28]. Yu et al. implemented auto-encoders for diagnosing faults in mechanical vibration signals [29]. Hadj-Attou et al. used gated recurrent units to analyze vehicle vibration signals for road condition assessment [30]. While these machine-learning methods provide deep feature extraction from vibration data, they come with high computational costs and time requirements. In road engineering, complex computations mean that monitored data are difficult to process in real time, resulting in delayed maintenance and incurring additional costs that increase the financial burden. For this study’s focus on efficient vibration analysis and vehicle detection on PCC pavement under dynamic loading, a method that allowed automated and real-time data processing was necessary. Here, the thresholding method presents a straightforward solution. Matsumoto et al. employed thresholding to study road traffic’s impact on building vibrations [31]. Zhang et al. used thresholding to correlate vehicle vibration with pavement roughness [32]. Qian et al. applied thresholding to assess the effect of airport runway roughness on aircraft vibrations [33]. Given its simplicity and wide applicability, this study adopted the thresholding method for analyzing vibration data collected by the MEMS accelerometer sensors.

In summary, this study was dedicated to the long-term monitoring of road performance and involved deploying MEMS accelerometer sensors on various slabs and locations within each slab of PCC pavement. A specialized thresholding method was then used to analyze the pavement’s acceleration time-domain signals under dynamic vehicle loading. Subsequently, stationary wavelet transform (SWT) was employed for denoising, which selectively eliminated noise while preserving the essential features of the acceleration signals. The goal was to identify how the vibration response magnitude varied across different pavement locations and to evaluate the effectiveness of vehicle detection. The study led to identifying optimal sensor placement for maximum vibration signal capture and optimal vehicle detection using as few sensors as possible. Compared with existing research, the novel contributions of this study included the following:

- Comparative analysis of the responses of different slabs and positions within slabs under dynamic vehicle loading, revealing front-end locations exhibited maximum acceleration amplitudes;
- Development of a thresholding method for processing acceleration time-domain signals from MEMS accelerometer sensors, achieving > 85% precision and recall without computationally intensive denoising processes;
- Determination of cost-effective sensor placement strategies, demonstrating slow-lane front-end positions achieved optimal F1 scores (>85%) with minimal sensor deployment.

## 2. Sensor Deployment

**Figure 1:**
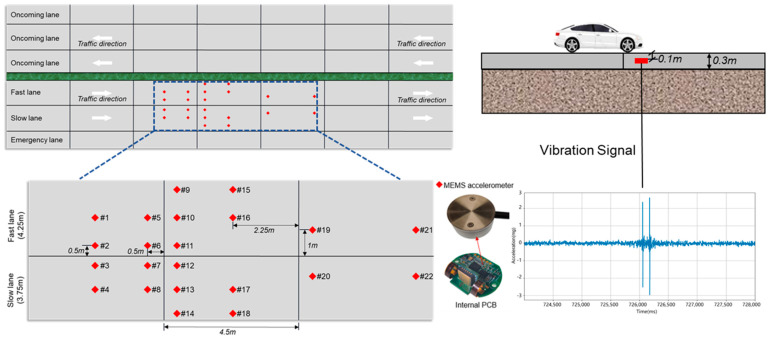
Deployment locations of MEMS accelerometer sensors to select the best location for detection.

In this research, 22 MEMS accelerometer sensors (Key Laboratory of Civil Engineering Safety and Durability of China Education Ministry, Tsinghua University, Beijing, China) were strategically deployed to assess the acceleration responses of various locations on PCC pavement under dynamic vehicle loading, as shown in Figure 1. The sensors were custom-designed, developed specifically for pavement monitoring applications. Key specifications included a resolution of 0.042 mg, a sampling frequency of 1000 Hz, a frequency monitoring range of 0.1–200 Hz, and a linearity of 1% across the measurement range. The operational temperature range spanned from −30 °C to +80 °C, ensuring reliability under diverse environmental conditions.

The MEMS accelerometer sensors were embedded within the PCC pavement structure, with their upper surfaces positioned 10 cm below the pavement surface. This depth selection aligned with findings by Ferreira et al. [34], who demonstrated that structural vibration signals, including peak acceleration and time-domain characteristics, exhibited negligible variation (coefficient of variation < 5%) across embedding depths ranging from 5 cm to 20 cm in concrete pavements. Additionally, the embedded configuration provided mechanical protection against vehicular abrasion and construction activities, further enhancing long-term monitoring reliability.

The study was conducted on a two-way, four-lane highway in ZhaoTong, Yunnan Province, China. This highway was constructed by initially paving the fast and slow lanes together, followed by longitudinal cutting along the lane markings. The emergency lane on the shoulder was paved separately. Additionally, the PCC pavement underwent lateral cutting, resulting in multiple slab formations.

Regarding the lateral placement of sensors, 11 sensors were installed in the fast lane, and another 11 in the slow lane. In the fast lane, two sensors were positioned on the left side (#9 and #15), four in the middle (#1, #5, #10, and #16), two on the right side near the middle (#19 and #21), and three on the extreme right (#2, #6, and #11). In the slow lane, three sensors were placed on the extreme left (#3, #7, and #12), two on the left side near the middle (#20 and #22), four in the middle (#4, #8, #13, and #17), and two on the right (#14, #18). The front end of each slab, where the vehicle first makes contact, was a key consideration in sensor positioning. Along the longitudinal axis, eight sensors were located at the front of slabs, eight in the middle, and six at the rear.

This sensor deployment strategy facilitated a comprehensive comparison of the acceleration signal responses at different positions when vehicles passed over, along with an evaluation of the vehicle-detection performance. The analysis aimed to establish a relationship between the acceleration responses and the different slabs of the PCC pavement under dynamic vehicle loading. This insight was crucial for determining the most effective sensor placement locations.

**Figure 2:**
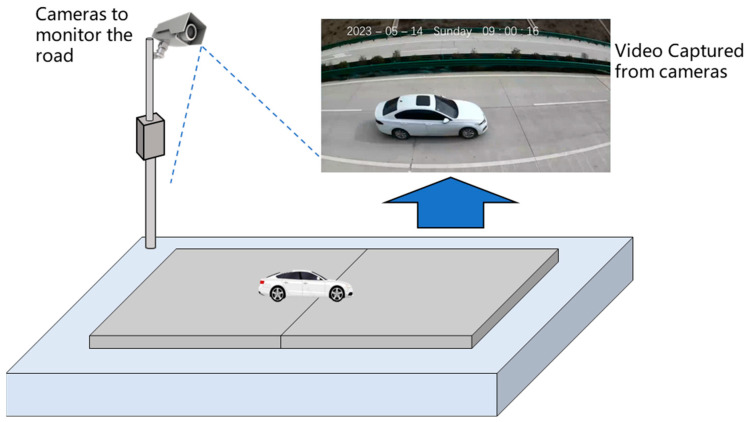
Real-world scenes of the road.

Additionally, video data were captured during on-road testing, as depicted in Figure 2**.** This manually recorded video data served as a benchmark, providing truth labels to validate the performance of the MEMS accelerometer sensors in vehicle detection.

## 3. Experimental Data and Processing

### 3.1. Data Preprocessing

The data gathered by the MEMS accelerometer sensors encompassed signal timestamps and magnitudes. Timestamps were logged every millisecond, while magnitudes were measured in milligravity (mg), equating to one-thousandth of the gravitational acceleration. In this context, 1 mg corresponded to an acceleration of 0.01 m/s^2^. Each recording session using these sensors lasted 1800 s, generating a substantial 1.8 million data points per data set. To make this voluminous data more manageable for analysis, preprocessing steps were undertaken.

(1) $$A_{i}=a_{i}-\frac{\sum_{i=1}^{n}a_{i}}{n}$$

where $A_{i}$ is magnitudes of the signals after centralization; $a_{i}$ is raw signal magnitudes; and *n* is number of signals in each data set, set at 1,800,000.

**Figure 3:**
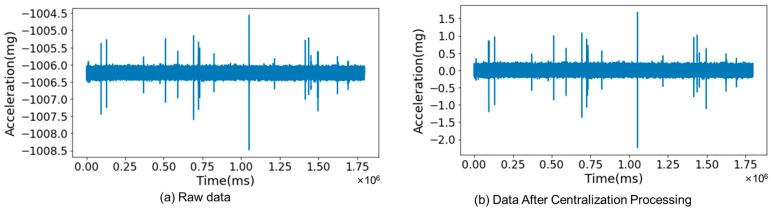
Centralization processing of raw data.

A feature of this preprocessing was the centralization of signal magnitudes. This involved adjusting each point in a data set by subtracting the average value of all points in that set, as described in Equation (1). Post-centralization, the mean of the acceleration signals was adjusted to 0. This process is visualized in Figure 3, where part (a) displays the original, raw signal data, and part (b) shows the data post-centralization.

Complementing the sensor data, manually recorded video data provided contextual details, including the timestamp of each vehicle’s passage relative to the sensor’s lateral road position, the lane of travel, and the vehicle type. The lane classifications used were the slow lane, fast lane, and oncoming lane. Vehicle types were categorized into cars and large vehicles. In this context, “cars” refers to passenger cars and other two-axle, four-tire single-unit vehicles, excluding trailers, while “large vehicles” refers to buses, trucks, and trailers, according to the Federal Highway Administration [35]. The monitoring period included several days in May 2023, during three daily time slots: from 9:00 AM to 9:30 AM, from 2:00 PM to 2:30 PM, and from 6:00 PM to 6:30 PM. During the monitoring period, the slow lane saw 242 cars and 35 large vehicles, the fast lane had 43 cars and 1 large vehicle, while the oncoming lanes were traversed by 260 cars and 34 large vehicles, as shown in Table 1.

### 3.2. Characteristics of Acceleration Response Signals on PCC Pavement Under Vehicle Loading at Different Time Scales

**Figure 4:**
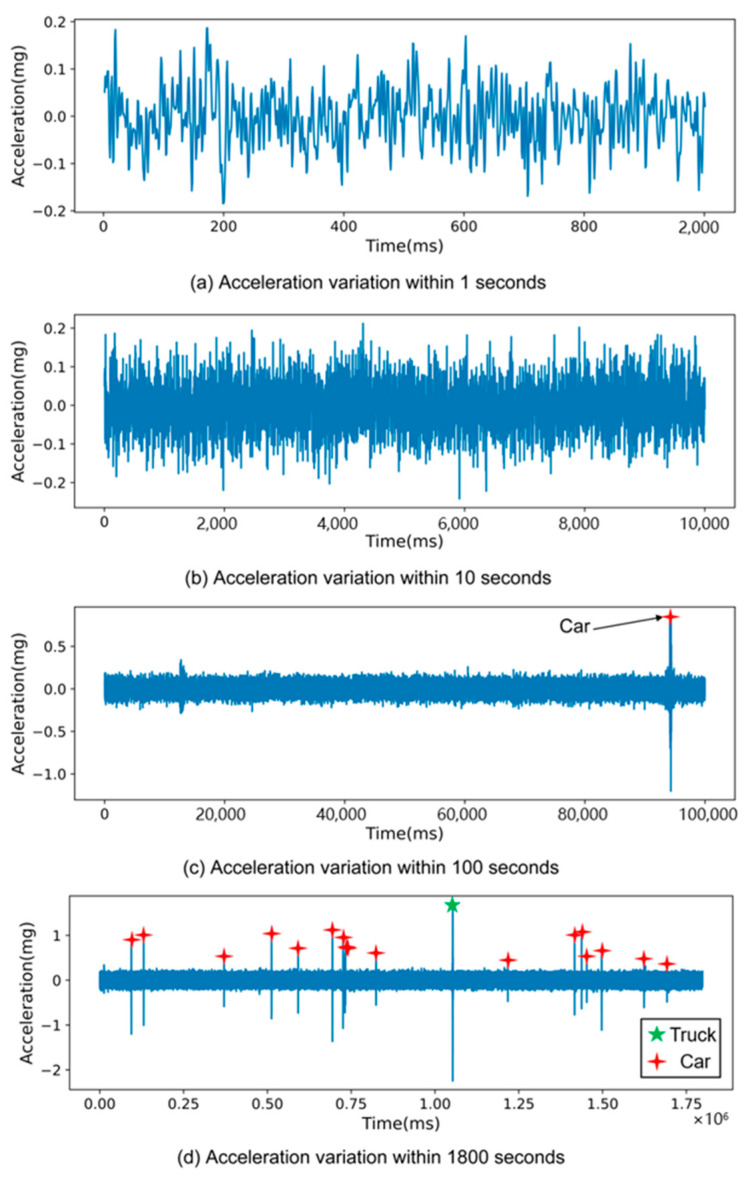
Characteristics of acceleration response signal of Sensor #4 under vehicle loading at different time scales.

This section delves into the distinct characteristics of the acceleration response signals on PCC pavement when subjected to vehicle loading across various time scales. For a detailed examination, Sensor #4—strategically placed in the middle of the slow lane and at the center of a slab—served as the focal point. Figure 4 graphically represents the acceleration response of this sensor over a thirty-minute period, illustrating responses at time scales of 1 s, 10 s, 100 s, and 1800 s.

In Figure 4a, the scenario without vehicle traffic is depicted. Here, the sensor’s acceleration response mainly exhibits irregular noise with a minimal amplitude. Moving to Figure 4b, the pattern remains largely consistent—predominantly small-amplitude, irregular noise dominates the signal.

The scenario changes notably in Figure 4c, where the presence of a vehicle passing over the sensor’s location on the pavement is observed. In this instance, the acceleration signal distinctly showcases peaks and valleys, indicating the influence of the vehicle’s weight on the pavement.

Further expanding the observation time in Figure 4d, it becomes evident that different vehicles created varying acceleration signal patterns. These variations in peak and valley amplitudes can be attributed to the differences in vehicle weight and the specific pressure points exerted by the vehicles on the pavement.

Analyzing the data presented in Figure 4, it is observed that the absolute values of acceleration signals representing noise generally do not surpass 0.2 mg. However, some instances do exceed this threshold. Consequently, when establishing a threshold for signal analysis, it is advisable to consider values distinctly above 0.2 mg to differentiate vehicle-induced signals from background noise effectively.

### 3.3. Data Denoising with SWT Method

Acceleration signal preprocessing through noise reduction constitutes a critical prerequisite for reliable vehicle detection. In this study, the stationary wavelet transform (SWT) was exclusively applied for denoising, without the use of additional filtering techniques. This choice was motivated by SWT’s inherent capability to handle non-stationary signals while preserving critical time-domain features of vehicle-induced vibrations, as demonstrated in prior studies [20,21].

Although ambient noise in acceleration measurements generally remains below 0.2 mg, transient noise exceeding this threshold can distort signal interpretation. Effective denoising must, therefore, eliminate extraneous noise components while preserving essential vehicle-induced acceleration features.

The non-stationary nature of pavement vibration signals further justifies the adoption of SWT. Traditional discrete wavelet transforms introduce Gibbs phenomena at signal discontinuities due to down-sampling. SWT overcomes this limitation through an interval zero-padding strategy that maintains coefficient dimensional consistency across decomposition levels. This approach preserves the original sampling resolution while suppressing aliasing artifacts.

The SWT-based denoising protocol implements three sequential stages:

(1) Decomposition:

A wavelet basis (e.g., Daubechies db6) and decomposition depth N are selected based on signal characteristics. The raw signal undergoes N-level decomposition, generating approximation coefficients (low-frequency components) and detail coefficients (high-frequency components) at multiple scales. Each decomposition level retains the original signal length.

(2) Threshold Processing:

The decomposed wavelet coefficients undergo threshold processing to separate pavement response components from noise. Pavement vibration-induced coefficients exhibit significantly higher magnitudes than noise-related components. A statistically derived threshold *λ* is applied as follows:

(2) $$Retained\;coefficient\;c_{i}=\{\begin{array}{c} c_{i},\;\;\left|c_{i}\right|\geq \lambda \\ 0,\;\;\left|c_{i}\right|<\lambda \end{array}$$

Threshold optimization critically balances denoising efficacy and feature preservation. Excessively high *λ* values risk attenuating genuine pavement responses, while insufficient thresholds permit residual noise contamination. The fixed thresholding method is commonly used for wavelet-based denoising, particularly for low-frequency signals, as it has been proven effective in various signal-processing applications [36]. The threshold is calculated using the following formulas:

(3) $$\lambda =\sigma \sqrt{2ln\left(N\right)}$$

(4) $$\sigma =\frac{midian\left(\left|y\right|\right)}{0.6745}$$

where *λ* represents the threshold of the wavelet coefficient, *N* denotes data length, *σ* represents noise standard deviation estimated via median absolute deviation, and *y* is the vector formed by the original signal.

(3) Reconstruction:

After thresholding, the signal is reconstructed using the inverse wavelet transform, which combines the processed approximation and detail coefficients to generate the denoised signal. This final signal retains the critical features of the original signal while minimizing the impact of noise. By following this approach, the SWT method effectively denoises the acceleration signal, ensuring that the key dynamic features caused by vehicle passage are preserved for subsequent analysis.

### 3.4. Acceleration Threshold Selection Based on Detection Performance

**Figure 5:**
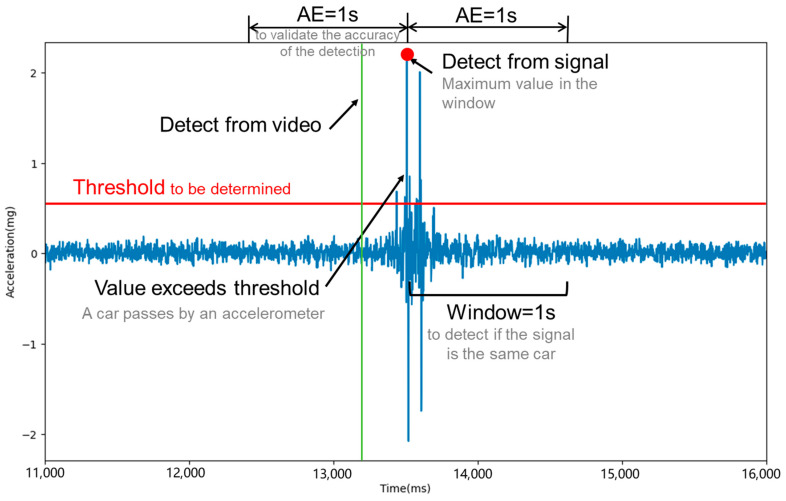
Schematic representation of vehicle detection using the thresholding method.

This section focuses on the implementation of a thresholding method using MEMS accelerometer sensors for detecting vehicles. After preprocessing the data as previously described, a specific threshold was established. Acceleration signals exceeding this threshold were then filtered, as shown in Figure 5. Given that acceleration signals were recorded every millisecond, and a vehicle took longer than this to pass over a sensor-equipped slab, multiple signals above the threshold could be generated by a single vehicle. To address this, a window-based approach was adopted.

(1) Synchronization of Video and Sensor Data

To ensure temporal alignment between manually recorded video data and sensor signals, a timestamp synchronization mechanism was implemented. The first five vehicles in each video were manually annotated with their passage timestamps. Simultaneously, the corresponding acceleration signal peaks for these vehicles were identified. The time difference $\Delta t_{i}$ between each vehicle’s video timestamp $T_{video,i}$ and the sensor-detected peak timestamp $T_{sensor,i}$ was calculated as follows:

(5) $$\Delta t_{i}=T_{video,i}-T_{sensor,i}\;\;\;\left(i=1,2,\ldots ,5\right)$$

The mean time offset $\bar{\Delta t}$ across these five vehicles was then computed:

(6) $$\bar{\Delta t}=\frac{1}{5}\sum_{i=1}^{5}\Delta t_{i}$$

This $\bar{\Delta t}$ was applied to shift the entire sensor timeline, aligning it with the video data. This method minimized systematic timing discrepancies caused by manual recording latency or sensor clock drift.

(2) Detection Methodology

In this approach, each time a signal surpasses the threshold, its timestamp is noted. If the timestamp of a subsequent signal that also exceeds the threshold is more than the designated window interval from the first, they are considered separate vehicle passages. If not, they are assumed to be from the same vehicle. This method detects entire vehicles rather than individual axles. For instance, a four-axle truck passing over a sensor-equipped slab may generate multiple peaks within a short interval. If each slab is 5 m long and vehicles typically travel faster than 5 m/s (18 km/h), it takes less than 1 s for a vehicle to traverse a slab. Conversely, two consecutive two-axle cars with a headway of >1 s would produce distinct clusters of signals, resulting in two separate vehicle detections. Therefore, the window was set at 1 s for this study, balancing the avoidance of misidentifying single vehicle passages as multiple ones and vice versa.

For cases where multiple signals from a single vehicle exceeded the threshold, the signal with the highest magnitude was chosen to represent that vehicle’s passage. The effectiveness of this threshold–window combination was verified by comparing the results with manually recorded video data labels. The allowable error (*AE*) is a critical factor here. If the time difference between the sensor’s identification and the video data timestamp was less than the *AE*, the identification was deemed accurate. The total number of correctly identified signals indicated the actual count of vehicles detected by the sensor. The *AE* must have been at least the longest of the label-recording interval, the sensor-recording interval, and the window interval, as detailed in Equation (2):

(7) $$AE\geq max\left(T_{label},T_{accelerometer},Window\right)$$

where *AE* is the allowable error; $T_{label}$ is label-recording interval, set at 1 s; $T_{accelerometer}$ is sensor-recording interval, set at 1 ms; and Window is window interval, set at 1 s.

For this study, the label-recording interval was 1 s, the sensor-recording interval was 1 ms, and the window interval was 1 s, necessitating an *AE* exceeding 1 s. Setting the *AE* to 1 s, as noise levels were substantially lower than the magnitudes of vehicle-induced signals, meant that detection outcomes were not affected. Furthermore, the proposed thresholding method, in conjunction with the window setting, effectively distinguished between single and multiple vehicle passages. Validation with video data confirmed that multi-axle vehicles (e.g., trucks) were consistently detected as single events, while consecutive vehicles were correctly separated.

(3) Quantitative Evaluation of Detection Performance

Based on the identification outcomes, the performance of the MEMS accelerometer sensor in detecting vehicles was evaluated by two key metrics: detection precision and detection recall. Detection precision is calculated as the ratio of the number of correctly identified vehicle passages to the total number of signals identified as vehicles exceeding the threshold. Detection recall, on the other hand, is the ratio of the number of correctly identified vehicle passages to the overall number of actual vehicle passages in the lane, as expressed in Equation (3).

(8) $$P=\frac{N_{A}}{N_{C}},\;R=\frac{N_{A}}{N_{T}}$$

where P is detection precision, R is detection recall, $N_{A}$ is the number of correctly identified vehicle passages, $N_{C}$ is the total number of signals identified as vehicles exceeding the threshold, and $N_{T}$ is the overall number of actual vehicle passages in the lane.

To assess the optimal balance between precision and recall, the harmonic mean of precision and recall, known as the *F_1_* score, was computed. The *F_1_* score provides a comprehensive measure of detection performance that considers both precision and recall. The formula for the *F_1_* score is given by

(9) $$F_{1}\text{-}score=\frac{2PR}{P+R}$$

where *P* is precision and *R* is recall.

**Figure 6:**
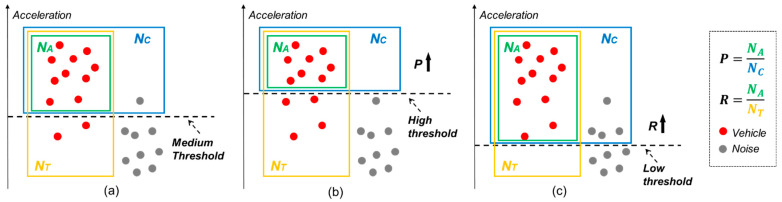
Relationship between the threshold and detection precision and recall. (**a**–**c**) The detection schematic with medium, high, and low thresholds.

Figure 6 demonstrates how the chosen threshold level impacts these metrics. A higher threshold generally reduces the sensor’s tendency to mistake noise for true signals, thereby improving detection precision. However, this also means that some genuine signals may be missed, lowering the detection recall. In contrast, a lower threshold increases the likelihood of noise influencing the results, decreasing precision. This approach captures a higher proportion of true vehicle signals, enhancing recall.

Therefore, the decision on threshold setting hinges on the specific goal of the vehicle-detection process. To prioritize identifying actual vehicle passages with greater accuracy, a higher threshold is advisable. But, if the objective is to detect as many vehicle passages as possible, a lower threshold will prove more effective, ensuring a better recall.

## 4. Analysis and Discussion

### 4.1. Vehicle-Detection Performance

The single sensor detection approach involves using data from just one sensor per lane for vehicle passage detection. This method’s appeal lies in its simplicity and cost-effectiveness. To address potential performance variations across sensor locations, each sensor was independently evaluated to determine its optimal detection threshold. The focus here was on selecting an appropriate threshold and determining the optimal sensor placement.

**Figure 7:**
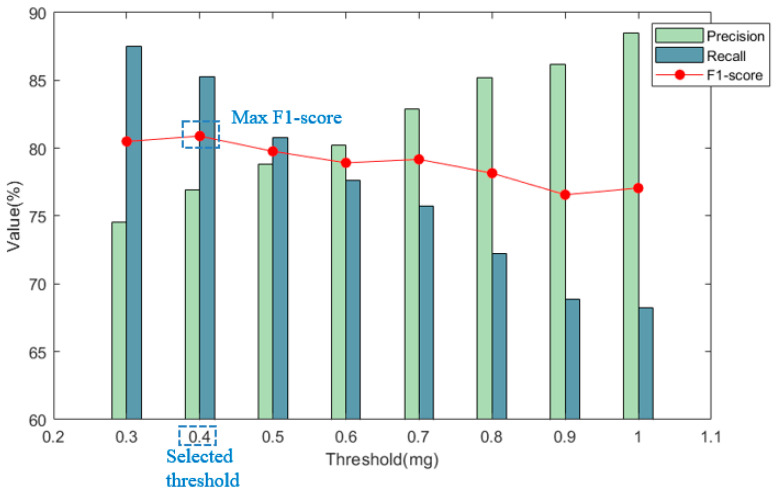
Detection precision, recall, and F1 score of Sensor #12, as an example, under different thresholds.

From the previous analysis, it was clear that since noise levels could surpass 0.2 mg, the threshold must be significantly higher. Thus, the minimum threshold was established at 0.3 mg. To investigate the effect of varying threshold values on vehicle-detection accuracy, eight different thresholds were tested: 0.3 mg, 0.4 mg, 0.5 mg, 0.6 mg, 0.7 mg, 0.8 mg, 0.9 mg, and 1.0 mg. Vehicle-detection performance was analyzed under these varying threshold conditions. The focus here was on selecting an appropriate threshold and determining the optimal sensor placement. Figure 7 presents the typical case of sensor # 12, demonstrating the method for selecting the optimal threshold.

Figure 7 illustrates the recall, precision, and F1 score of sensor #12 under eight different thresholds ranging from 0.3 to 1.0, as detected by the threshold detection method. It can be observed from the figure that as the threshold increased, the precision gradually increased, while the recall rate gradually rose. To balance both recall and precision, the F1 score was chosen as the comprehensive evaluation criterion for the detection effectiveness. The threshold corresponding to the maximum F1 score was deemed the optimal threshold. The F1 score of each sensor under the optimal threshold indicated the best detection effect of the position sensor. By comparing the optimal F1 score of different position sensors, the largest F1 score was selected, and the corresponding sensor position was the best placement position of the sensor in vehicle detection. Table 2 lists the optimal thresholds for the 22 sensors determined by the aforementioned method.

**Figure 8:**
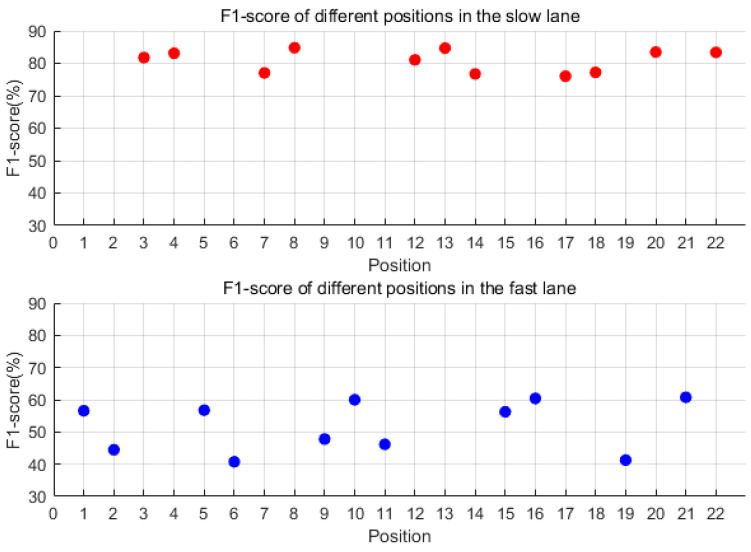
The F1 score of each of the 22 sensors under the optimal threshold. Red dots represent F1-scores for the slow lane; blue dots represent F1-scores for the fast lane.

For each of the 22 sensors, individualized optimal thresholds (as listed in Table 2) were determined through exhaustive evaluation across eight candidate threshold values (0.3–1.0 mg). Each sensor’s F1 score was calculated based on its own optimal threshold, ensuring that performance metrics reflected the tailored adaptation to localized signal characteristics and noise conditions. Figure 8 visualizes these sensor-specific F1 scores, with red and blue markers distinguishing slow-lane (n = 11) and fast-lane (n = 11) sensors, respectively. Analysis revealed significantly superior detection performance in the slow lane, where F1 scores ranged from 75% to 85% compared with 40–60% in the fast lane. Among slow-lane sensors, #8 achieved peak performance with an F1 score of 84.79% (precision = 83.9%, recall = 85.7%) at a 0.4 mg threshold. Three additional sensors (#20, #22, and #13) exceeded 83% F1 score, suggesting these four positions as optimal deployment locations. The performance gap between lanes demonstrates a 35–45% relative improvement in slow-lane detection capability, highlighting the critical influence of traffic flow characteristics on sensor effectiveness.

The elevated traffic density in slow and oncoming lanes induced frequent threshold exceedances in fast-lane sensors, resulting in a 42% increase in the false-positive rate compared with controlled conditions. In contrast, slow-lane sensors maintained detection precision exceeding 70%, demonstrating superior reliability under actual traffic flow conditions. This performance disparity stems from two key factors: (1) low heavy-vehicle proportion in slow lanes minimizes axle-load-induced signal saturation and (2) increased lateral separation from oncoming traffic attenuates cross-lane interference signals.

To summarize, when using a single sensor approach, the best compromise between precision and recall was achieved with a 0.4 mg threshold. The ideal sensor locations were in the front and back end of the slow lane, particularly at positions #8, #13, #20, and #22. In such a setup, the F1 score can surpass 85%, and precision and recall can surpass 80%, offering a balanced approach to vehicle detection.

### 4.2. The Impact of Signal Denoising Based on SWT for Single Sensor

In the previous section, this paper details analysis of the impact of different threshold selections on the sensor’s recall and precision rates at various positions on the road. In this section, the paper elaborates further on the changes in the performance of individual sensors for vehicle detection after applying a signal denoising method based on stationary wavelet transform (SWT). The primary objective of this paper was to evaluate whether noise-reduction processing could improve the sensor’s recall and precision rates at different positions on the road.

**Figure 9:**
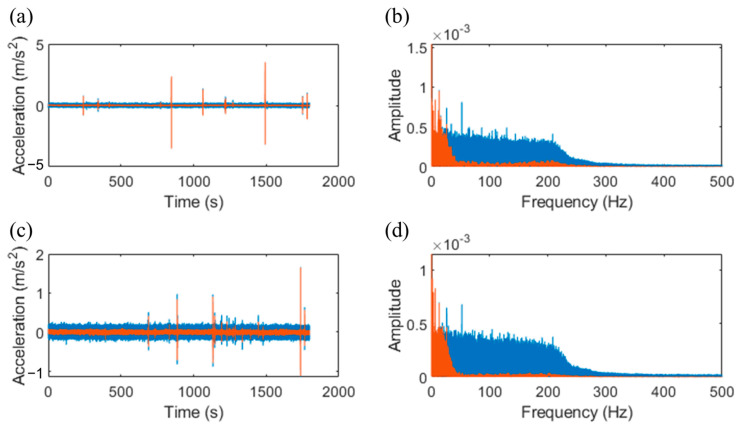
Acceleration signal before and after denoising. The blue curve is the original signal, and the red curve is the signal after denoising. (**a**,**c**) The time-domain vibration signal and (**b**,**d**) the frequency-domain vibration signal, respectively.

Figure 9 presents the comparison of acceleration signals before and after denoising. The denoising process, based on SWT, effectively reduced high-frequency noise while preserving the key features of the original signal. In the time domain (Figure 9a,c), it is evident that the denoised signal retained the main characteristics of the vehicle-induced vibrations, while the noise level was significantly reduced. The maximum noise level before denoising was approximately 0.2 mg, whereas the post-denoising signal exhibited a maximum noise value of 0.06 mg. Figure 9b,d shows the frequency-domain signal before and after denoising. After using the wavelet transform denoising method, the high frequency component of the vibration signal was significantly reduced. Compared with the vehicle signal, the noise signal had a higher content of high-frequency components, so it can be considered that the wavelet transform denoising method can effectively remove the noise components in the vibration signal. This lays the foundation for subsequent vehicle inspection and analysis, helping to improve overall system performance and reliability.

Following the denoising process, vehicle detection was performed using the same thresholding method as in previous sections. Table 3 lists the optimal thresholds for the 22 sensors calculated from the denoised data.

**Figure 10:**
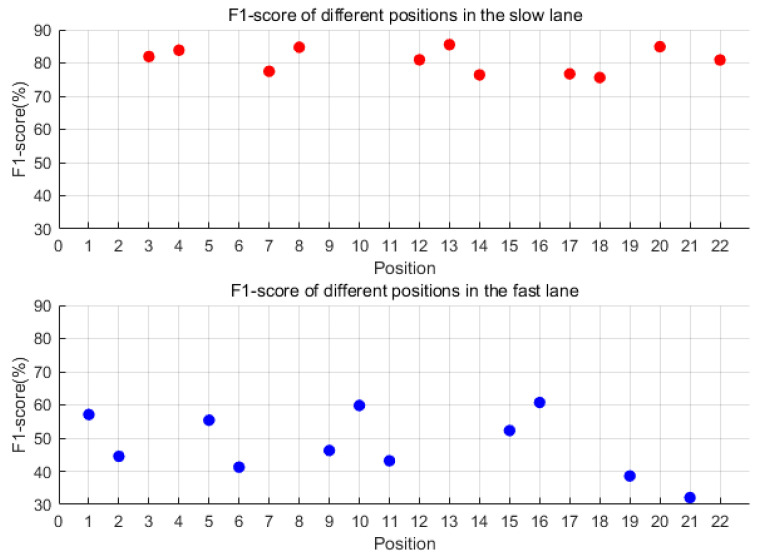
The F1 score of each of the 22 sensors under the optimal threshold after denoising. Red dots represent F1-scores for the slow lane; blue dots represent F1-scores for the fast lane.

For each of the 22 sensors, individualized optimal thresholds (as listed in Table 2) were determined through exhaustive evaluation across eight candidate threshold values (0.3–1.0 mg). Each sensor’s F1 score was calculated based on its own optimal threshold, ensuring that performance metrics reflected the tailored adaptation to localized signal characteristics and noise conditions. Figure 10 visualizes these sensor-specific F1 scores, with red and blue markers distinguishing slow-lane (n = 11) and fast-lane (n = 11) sensors, respectively. As shown in Figure 10, the denoising process showed negligible variation in detection performance between lanes (ΔF1 < 1.2%). Slow-lane sensors (red markers) maintained superior performance, with Sensor #13 achieving peak metrics (F1 = 85.5%, precision = 85.5%, recall = 85.6%) at 0.3 mg threshold. Two additional slow-lane sensors (#8 and #20) exceeded 84% F1 score, identifying four optimal deployment positions. Fast-lane sensors (blue markers) demonstrated limited improvement (F1 < 62%), revealing fundamental constraints of the denoising methodology.

The observed performance limitation stems from spectral overlap between target signals and noise components. SWT processing removes high-frequency components; however, it inadvertently suppresses some of the lower-amplitude vehicle-induced vibrations, especially those from smaller vehicles. This suppression can lead to misclassification of weaker vehicle signals as noise, resulting in decreased detection reliability for smaller vehicles, which are more prevalent in the fast lane.

The results suggest that while SWT denoising effectively reduced high-frequency noise, it does not significantly enhance vehicle-detection performance in this study: time-domain detection F1 score increased from 84.8% to 85.5%, by only 0.7%. The attenuation of weak vehicle signals during denoising may offset the benefits of noise reduction, particularly for smaller vehicles. Therefore, in this context, the application of denoising does not substantially improve detection accuracy, and future research should investigate other noise-reduction techniques or explore alternative approaches to vehicle detection that do not rely heavily on signal denoising.

### 4.3. Vibration Response and Cross-Lane Interference Analysis of Slabs Under Vehicle Dynamic Load

When vehicles traverse PCC pavement, distinct acceleration responses are observed at various positions, reflecting the pavement’s service performance and structural health. To explore the link between different pavement positions’ responses to dynamic vehicle loads and to deploy sensors cost-effectively, an analysis of vibration responses of each slab and position under dynamic loading was necessary. This analysis further evaluated cross-lane vibration interference and multi-vehicle scenarios to validate detection robustness.

A threshold of 0.4 mg was utilized for this analysis. For each MEMS accelerometer sensor, every detected signal was recorded. The average magnitude of acceleration response under dynamic vehicle load for each sensor was calculated, as outlined in Equation (4):

(10) $$A=\frac{\sum_{i=1}^{N_{C}}AC_{i}}{N_{C}}$$

where A is the average magnitude of acceleration response under vehicle dynamic load and is represented for that sensor, $AC_{i}$ is the detected signals exceeding the threshold by the sensor, and $N_{C}$ is the total number of signals identified as vehicles exceeding the threshold.

**Figure 11:**
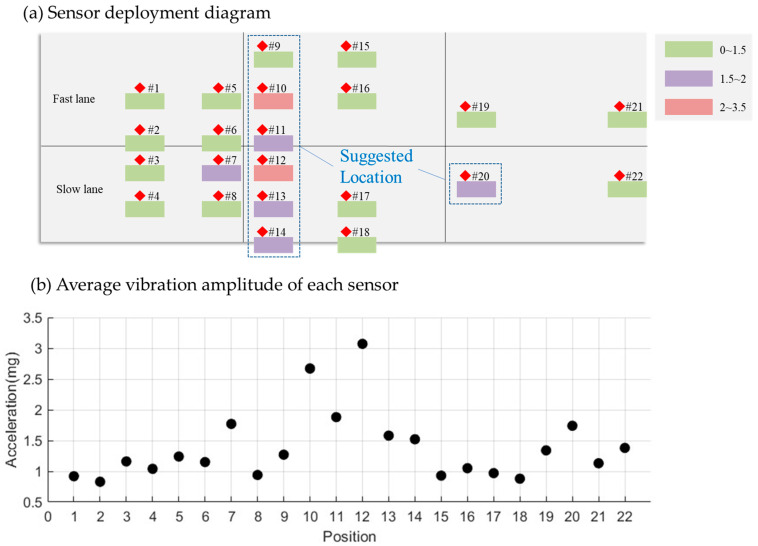
Average vibration amplitude of each sensor for all cars that passed by.

As seen in Figure 11, in the fast lane, Sensor #10 registered the highest average acceleration signal at 2.67 mg. In the slow lane, Sensor #12 recorded the highest average acceleration signal, measuring 3.07 mg. A comparison of signal responses across different sensor positions reveals that sensors at the front of the slab exhibited the strongest response, followed by those at the rear, while mid-slab sensors showed the weakest response. Consequently, it is advisable to place sensors at the slab’s front, as marked by the blue frame in Figure 11.

**Figure 12:**
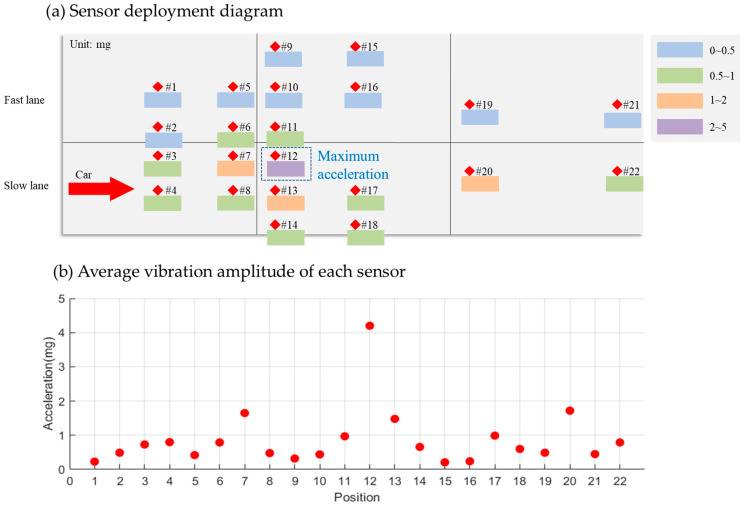
Average vibration amplitude of each sensor for the cars passing through the slow lane.

Focusing on vibration signals caused by slow-lane vehicles, one data set that shows the maximum acceleration signal generated by each sensor is displayed in Figure 12. Here, Sensor #12, located at the slab’s front end, showed the strongest response at 4.20 mg. Sensors #20 and #13, also at the front, and Sensor #7 at the rear, registered responses between 1 mg and 2 mg. Responses from other sensors fell below 1 mg. Notably, the average acceleration amplitude of fast-lane sensors induced by slow-lane vehicles was 0.42 mg ($\sigma^{2}$ = 0.03 mg), below the detection threshold of fast-lane sensors, that ranged from 0.7 mg to 1.0 mg, confirming minimal cross-lane interference.

**Figure 13:**
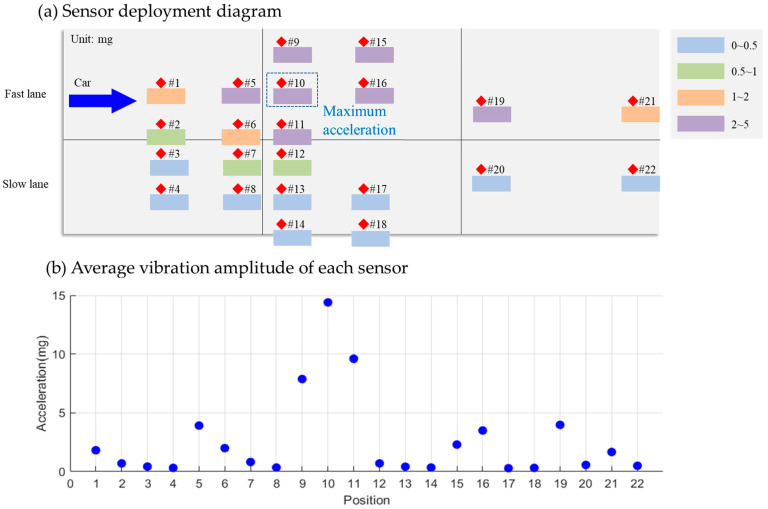
Average of vibration amplitudes of each sensor for the cars passing through the fast lane.

Focusing on vibration signals caused by fast-lane vehicles, as illustrated in Figure 13, Sensor #10 at the slab’s front end recorded the highest response at 14.4 mg for a specific data set. Sensors #9 and #11, also at the front, showed significantly higher responses (7.62 mg and 9.65 mg, respectively) compared with other locations. Concurrently, slow-lane sensors demonstrated average amplitudes of 0.40 mg ($\sigma^{2}$ = 0.02 mg) during fast-lane vehicle passages (Figure 13b), below the detection threshold of fast-lane sensors that ranged from 0.4 mg to 0.5 mg. This contrast reaffirms that sensors at the slab’s front end yielded the maximum acceleration response while cross-lane interference remained insignificant.

In summary, sensors positioned at the front end of cement concrete slabs consistently exhibited the most intense signal responses when vehicles passed over. For enhanced vibration signal analysis and increased detection accuracy, strategically placing sensors at these front-end positions (such as #12, #13, and #20) is recommended.

## 5. Conclusions

This study was centered around understanding how different slabs and positions on PCC pavement respond to dynamic vehicle loading and the development of an automated, precise, and cost-effective vehicle-detection method. The key conclusions from our analyses and proposals for sensor deployment strategies are as follows:

1. Thresholding method for vehicle detection: A novel approach was designed using MEMS accelerometer sensors to detect vehicles on PCC pavement. This method relies on time-domain acceleration signals and offers rapid and accurate vehicle detection capabilities.
2. The effect of denoising processing on signal detection: The application of SWT-based denoising did not result in significant improvements in detection performance. While denoising reduced noise, it also attenuated weaker vehicle signals, thereby reducing detection reliability for smaller vehicles. Therefore, effective vehicle detection can be achieved without this additional processing.
3. Analysis of acceleration responses at different positions: The study revealed that the strongest MEMS accelerometer sensor signals occurred at the front end of the slabs, followed by the rear end, and were weakest in the middle. Therefore, placing sensors at the front end of the slab is suggested for capturing maximum acceleration responses, typically exceeding 2 mg due to vehicle loading.
4. Optimal sensor deployment for vehicle detection: For enhanced detection accuracy on PCC pavement, specific sensor placement strategies are recommended. Placing a sensor on front end or rear end of the slow lane can result in a sensor accuracy and recall rate of more than 85%.

## 6. Future Work

While this study focused on vehicle detection through acceleration signal analysis, the collected data and methodologies lay a foundation for several promising research directions:

- Vehicle parameter estimation: The integration of machine-learning techniques with acceleration signals could enable the intelligent detection of vehicle parameters such as weight and speed. By correlating time-domain vibration patterns (e.g., peak amplitude, signal duration, and frequency spectra) with vehicle characteristics, dynamic models could be developed to infer axle configurations and load distributions.
- The sensor network could be extended to detect early-stage pavement damage, such as cracking or settlement. By analyzing long-term vibration data for anomalies in signal energy, frequency shifts, or spatial response patterns, predictive maintenance models could identify localized structural degradation.

## Figures and Tables

**Table 1:** Deployment locations of MEMS accelerometer sensors.

	Cars	Large Vehicles	Total
Slow lane	242	35	277
Fast lane	43	1	44
Oncoming lanes	260	34	294
Total	545	70	615

**Table 2:** The selected threshold for each sensor. The red-marked font represents optimal deployment locations and the corresponding threshold.

Sensor Position	Selected Threshold (mg)	Sensor Position	Selected Threshold (mg)
1	0.7	12	0.5
2	0.7	13	0.4
3	0.4	14	0.3
4	0.4	15	1.0
5	0.9	16	0.9
6	0.8	17	0.4
7	0.4	18	0.3
8	0.4	19	1.0
9	1.0	20	0.4
10	0.9	21	0.3
11	1.0	22	0.5

**Table 3:** The selected threshold for each sensor after denoising. The red-marked font represents optimal deployment locations and the corresponding threshold.

Sensor Number	Selected Threshold (mg)	Sensor Number	Selected Threshold (mg)
1	0.6	12	0.4
2	0.6	13	0.3
3	0.3	14	0.3
4	0.3	15	0.9
5	0.8	16	0.8
6	0.9	17	0.3
7	0.3	18	0.3
8	0.3	19	0.9
9	1.0	20	0.4
10	0.9	21	0.7
11	1.0	22	0.4

## Data Availability

Data are contained within the article
